# Exosomes produced from 3D cultures of umbilical cord mesenchymal stem cells in a hollow-fiber bioreactor show improved osteochondral regeneration activity

**DOI:** 10.1007/s10565-019-09504-5

**Published:** 2019-12-09

**Authors:** Litao Yan, Xing Wu

**Affiliations:** Department of Orthopedics, Shanghai Tenth People’s Hospital, School of Medicine, Tongji University, Shanghai, 200072 People’s Republic of China

**Keywords:** Osteochondral regeneration, Umbilical cord mesenchymal stem cell, Exosome, 3D culture

## Abstract

Animal and clinical studies have shown that mesenchymal stem cells (MSCs) play an important role in cartilage repair. The therapeutic effect of mesenchymal stem cells based therapies has been increasingly demonstrated to exosome-mediated paracrine secretion. Here, we investigated the cellular processes and mechanism of exosomes produced by conventional 2D culture (2D-Exos) and exosomes produced from 3D culture (3D-Exos) of umbilical MSCs (U-MSCs) in a hollow-fiber bioreactor for the treatment of cartilage repair. We found that the yield of 3D-Exos was 7.5-fold higher than that of 2D-Exos. The in vitro experiments indicated that both 2D-Exos and 3D-Exos can stimulate chondrocyte proliferation, migration, and matrix synthesis, and inhibit apoptosis, with 3D-Exos exerting a stronger effect than 2D-Exos. This effect was partly attributed to the activation of transforming growth factor beta 1 and Smad2/3 signaling. The injection of 2D-Exos and 3D-Exos showed enhanced gross appearance and attenuated cartilage defect; however, 3D-Exos showed a superior therapeutic effect than 2D-Exos. In summary, our study provides novel insights into the chondroprotective effects of exosomes produced from 3D culture of U-MSCs in a hollow-fiber bioreactor. Because of its promising biological function and high yield, 3D-Exos may become a promising therapeutic method for the treatment of cartilage defects.

## Introduction

The adult hyaline articular cartilage is an avascular tissue that exhibits limited self-renewal capacity (Chijimatsu and Saito 2019). Traditional treatment modalities such as autografting, autologous chondrocyte implantation, and micro-fracture can temporarily alleviate symptoms, but often fail to fully restore the damaged cartilage because the regenerated tissue is unable develop an organized tissue architecture with surrounding hyaline cartilage (Gao et al. 2018).

In recent years, therapies based on stem cells have led to promising approaches for cartilage repair (Medvedeva et al. 2018; Patel et al. 2019). Laboratory and clinical studies have shown that mesenchymal stem cells (MSCs) can differentiate into chondrocytes and restore cartilage defects (Huang et al. 2016; Driessen et al. 2017; Ha et al. 2019). However, the potential of MSCs is hampered by aberrant cell phenotype, poor self-renewal ability, and reduced ability to differentiate after multiple passages when cultured in vitro. For transplantation of MSCs into the defect, cell vitality and viability need to be preserved; however, this requires proper handling and storage of cells and is logistically challenging (De Bari and Roelofs 2018; Dubey et al. 2018). After transplantation, the newly formed cartilage can encounter undesired hypertrophy and ossification, both of which can complicate clinical treatment (Music et al. 2018). Several studies have found that the curative effect of MSCs is more likely attributed to paracrine secretion of trophic factors rather than their differentiation into chondrocytes. Among these paracrine factors, exosomes are the most prominent extracellular vesicles that can mediate osteochondral regeneration. Exosomes are bilipid and nano-sized (50–150 nm) vesicles that contain nucleic acids and proteins (Toh et al. 2017). Zhang et al. (2016) have demonstrated that exosomes derived from MSCs can repair and regenerate osteochondral defects when intra-articular injected in a rat. Moreover, histological scores of the gross specimen in exosome-treated defects were significantly higher than those of control groups in vivo.

This study suggested the effectiveness of exosomes for cartilage repair, and it inspired us the cell-free therapeutic alternative to cell-based MSC therapy.

MSCs cultured in two-dimensional (2D) conventional tissue culture polystyrene flasks can result in extremely low yields, which can limit its clinical application. In our previous study, we have demonstrated that MSCs can promote the generation of cartilage and inhibit cartilage aging and osteogenesis when cultures in a 3D microgravity environment (Liu et al. 2016). We hypothesized that 3D culture might be an important factor for improved biological function. Haraszti et al. (2018) confirmed our hypothesis when they successfully obtained scalable 3D microcarrier-based cultures of MSCs. The effect of exosomes in small interfering RNA transferring was 7-fold than 2D culture. Therefore, we speculated that exosomes produced from MSCs by 3D culture might play an important role in osteochondral regeneration. They (Haraszti et al. 2018) also found that U-MSCs yielded four times as many exosomes per cell than did MSCs from bone marrow or adipose tissue.

Until now, a commercial hollow-fiber bioreactor system has been utilized effectively for large-scale exosome production (Yan et al. 2018). In this bioreactor, cells are seeded into cylindrical fibers to simulate 3D culture. Watson et al. (2016) obtained 5-fold more exosomes using circulating medium compared to those obtained from the conventional 2D cell culture. This bioreactor not only allows 3D culture, but also increases the yield of exosomes. Thus, we used this bioreactor to simulate 3D culture of U-MSCs and compared the efficacy of the exosomes produced by this method with those obtained from the conventional 2D flask culture on osteochondral regeneration.

## Methods

### Derivation of chondrocyte

Specimens of adult articular cartilage samples were isolated from patients undergoing total knee replacement surgery. Briefly, the cartilage sample was cut into pieces about 1 mm^3^ and treated with 0.5% collagenase II for 4 h. The mixture was filtered through the 100-μm cell strainer and transferred to monolayer culture in DMEM-F12 (Gibco, Grand Island, NY, USA) containing 10% fetal bovine serum (FBS) and 1% penicillin/streptomycin. After passage 3, chondrocytes were used for further in vitro experiments.

### 3D culture of U-MSCs and isolation of U-MSCs-Exos

The primary umbilical cord mesenchymal stem cell (ASNCUC1801305, SNC, Shanghai, China) was expanded in polystyrene flasks and then seeded into a medium-sized, hollow-fiber culture cartridge. The bioreactor was purchased from FiberCell Systems (C2025, Frederick, MD, USA), and the fiber surface area was 80 cm^2^. The exosomes secreted by cells were gathered in the extracellular space, which had a volume of 3.9 mL. The number of cell in 3D or 2D culture was all controlled to 1 × 10^7^ (almost ten T25 culture flasks). The cell density of 3D culture is 2.5 × 10^6^ cells/mL. Bioreactor conditioned medium was collected for every other day. The FBS used for culture of U-MSCs was centrifuged at 110,000*g* overnight to deplete exosomes. The protocol for the purification of exosomes was accorded to Théry et al. (2018). Supernatants collected from bioreactor or conventional culture flask were centrifuged at same speeds (3000*g* for 15 min; 20,000*g* for 45 min). Supernatants were passed through 0.22-μm filter and centrifuged for 70 min at 110,000*g* to pellet exosomes. The pellets were resuspended in 5 mL PBS and centrifuged for another 70 min at 110,000*g*. All purification steps were performed at 4 °C. Final pellets of exosomes were suspended in PBS and stored at − 80 °C. The Bradford method (Bio-Rad, Hercules, USA) was used to quantify the protein concentration of the exosomes.

### Transmission electron microscopy

Exosomes were suspended in PBS and dropped on the copper grid. After the pallets were dried in the air, they were fixed with 3% (w/v) glutaraldehyde for 2 h and negatively stained with 2% uranyl acetate for 30 s. The samples were observed using transmission electron microscope.

### Western blot analysis

Specific markers of exosomes (CD63(ab59479), CD81(ab79559), TSG101(ab125011)) and negative marker (Calnexin (ab 92573)) were tested by western blotting. Exosomes were lysed using RIPA and heated at 95 °C for 5 min with protein loading buffer, followed by subjecting to 12% SDS-PAGE polyacrylamide gels for 45 min and transferred to a PVDF membrane for 60 min. After incubating in BSA, PVDF membranes were incubated respectively with primary antibodies at 4 °C overnight. Membranes were washed with PBST for three times, and then incubated with appropriate secondary antibody for 1 h. All membranes were developed by enhanced chemiluminescence substrate.

### Nanoparticle tracking analysis

Size distribution of exosomes was measured using ZetaView instrument. Samples were diluted 1000-fold with PBS and added to the nanopores for measurement. Readings were performed with a particle analyzer.

### Chondrocyte proliferation assay (Cell Counting Kit-8)

The Cell Counting Kit-8 assay (Beyotime, Shanghai, China) was carried out to investigate the effect of 2D-Exos and 3D-exos in facilitating chondrocytes proliferation. Chondrocytes (2 × 10^3^ cells/well) pretreated with 10 μg/mL of the two different exosomes were seeded on 96-well cell plates. The medium was changed daily for 4 days. Standard curves were constructed by measuring optical density (OD) value at 450 nm.

### Cell apoptosis assay

The chondrocyte apoptosis was detected by V-FITC/PI according to the instructions (Beyotime, Shanghai, China). Briefly, chondrocytes at density of 5 × 10^5^ cells/mL were incubated with 10 μg/mL 2D-Exos and 3D-Exos, followed by 10 ng/mL IL-1β challenge for 24 h. Samples were harvested and washed with PBS for three times, and then resuspended in the binding buffer. A total of 5 μL Annexin V-FITC and 5 μL PI were added into the suspension. After incubation away from light for 15 min, mixtures were analyzed using flow cytometry (BD Biosciences).

### Chondrocyte scratch wound assay

The migration of chondrocytes was detected by scratch wound assay. Briefly, 4 × 10^4^ cells were covered the bottom of a 6-well plate. P200 pipet tip was used to scratch across the confluent monolayer of chondrocytes. The cell debris was washed with PBS. Then 1% FBS DMEM-F12 with 10 μg/mL exosomes was added. The condition of migration was captured at the same position after 0, 24, and 48 h.

### In vitro chondrocyte transwell migration assay

The chemotactic response of chondrocyte with various exosomes was evaluated using a transwell assay (Sigma). Chondrocyte were starved in serum-free medium for 24 h and seeded into the upper chambers (2 × 10^5^ cells). Then 5 μg exosomes with 500 μL 1% FBS DMEM-F12 were added to the lower chambers. After incubating at 37 °C for 12 and 24 h, chondrocytes under the insert filter were fixed and then stained with 0.1% crystal violet.

### Sulfated glycosaminoglycan and DNA quantification

Sulfated glycosaminoglycan was measured by Blyscan™ Glycosaminoglycan Assay following manufacturer’s instructions. Briefly, 1.0 mL Blyscan was added and spinned at 12,000 rpm for 10 min. The precipitate was mixed with dissociation reagent (0.5 mL). All samples, standards and blanks were measured using a microplate reader at 656 nm. DNA was measured was measured by Quant-iT™ Picogreen dsDNA Reagent and Kits. Briefly, 1.0 mL aqueous working solution was added and incubated for 5 minutes avoiding light. Samples were measured by the fluorescence microplate reader.

### Western blot

After 3 days of stimulation (10 μg/mL exosomes), chondrocytes were lysed using RIPA and heated at 95 °C for 5 min with protein loading buffer, followed by subjecting to 6–15% SDS-PAGE polyacrylamide gels for 45 min and transferred to a PVDF membrane for 60 min. After incubating in BSA, PVDF membranes were incubated respectively with primary antibodies at 4 °C overnight. Membranes were washed with PBST for three times, and then incubated with appropriate secondary antibody for 1 h. Glyceraldehyde 3-phosphate dehydrogenase (GAPDH) was chosen as the internal reference. All membranes were developed by enhanced chemiluminescence substrate. The detail information of antibodies was in Table 1.

**Table 1:** The detailed information of antibodies

Antibody	Product code	Company	Polyclonal or monoclonal
CD63	ab59479	abcam	Monoclonal
CD81	ab79559	abcam	Monoclonal
TSG101	ab125011	abcam	Monoclonal
Calnexin	ab92573	abcam	Monoclonal
CD9	ab92726	abcam	Monoclonal
Alix	ab186429	abcam	Monoclonal
GAPDH	ab181602	abcam	Monoclonal
PCNA	ab92552	abcam	Monoclonal
Survivin	ab76424	abcam	Monoclonal
Bcl-2	ab32124	abcam	Monoclonal
Bax	ab32503	abcam	Monoclonal
PDGFA	bs-0196R	Bioss	Polyclonal
FGF-2	bs-0217R	Bioss	Polyclonal
Col II	NB600-844	novus	Monoclonal
Aggrecan	WL02316	Wanleibio	Polyclonal
Sox9	ab185966	abcam	Monoclonal
MMP13	ab39012	abcam	Polyclonal
ADAMTS5	bs-3573R	Bioss	Polyclonal
TGFB1	A0291	Abclonal	Monoclonal
Smad2/Smad3	A7536	Abclonal	Polyclonal
phospho-Smad2/Smad3	bs-8853R	Bioss	Polyclonal
Runx2	A2851	Abclonal	Polyclonal

### Quantitative RT-PCR analysis

After 3 days of stimulation (10 μg/mL exosomes), total RNA of chondrocytes was extracted by the manufacturer’s instructions. PrimeScript RT-PCR kit (TaKaRa, China) was used for reversed transcription of 2 μg RNA for cDNA. RT-PCR reactions were performed at the following conditions: 95 °C for 3 min, 40 cycles of 95 °C for 3 s, 60 °C for 30 s. Beta-2-microglobulin was used as an internal reference to standardize the expression of other mRNAs. The result of target gene expression was calculated by the 2^–ΔΔCT^ method expressed as the mean of three samples and presented as fold increases relative to the negative control. All primer sequences used in this study are listed in Table 2.

**Table 2:** Primer sequences for quantitative real-time polymerase chain reaction

Gene	Primer	Sequence(5′–3′)
Aggrecan	Forward	ACCAGACTGTCAGATACCCC
	Reverse	CATAAAAGACCTCACCCTCC
Sox9	Forward	GCCTCTACTCCACCTTCACC
	Reverse	GTAGACGGGTTGTTCCCAGT
COL II	Forward	ATTGCCTATCTGGACGAAGC
	Reverse	GCAGTGTACGTGAACCTGCT
MMP-13	Forward	GCATTGGCTGAGTGAAAGAGAC
	Reverse	ATGATGAACGATGGACAGATGA
Runx2	Forward	ATGATTCGCCTCGGGGCTC
	Reverse	GCACTCTCCGAAGGGGATCT
PCNA	Forward	ATGATTCGCCTCGGGGCTC
	Reverse	GCACTCTCCGAAGGGGATCT
ADAMTS5	Forward	ATGATTCGCCTCGGGGCTC
	Reverse	GCACTCTCCGAAGGGGATCT
Survivin	Forward	TTCAAGGAGCTGGAAGGCTG
	Reverse	GCATCCGGACGAATGCTTTT
BCL2	Forward	TCTCATGCCAAGGGGGAAAC
	Reverse	CAATCCTCCCCCAGTTCACC
Bax	Forward	TCAAACCCTGCCCGAAACTT
	Reverse	TCAGATGCCGAAGTGTGTCC
FGF-2	Forward	GCTGTACTGCAAAAACGGGG
	Reverse	AGCCAGGTAACGGTTAGCAC
PDGF-AA	Forward	GGTCGCTCCTGAAGCCAG
	Reverse	GGAGGAGAAACAGGGAGTGC
Beta-2-microglobulin	Forward	ATCTGAGCAGGTTGCTCCAC
	Reverse	GGCCCTTTACACTGTGAGCC

### In vivo rabbit cartilage defect model and histological evaluation

Fifteen 2-kg New Zealand rabbits were randomly divided into three groups. The Drill bit (4-mm diameter) was used to operate cartilage defects on the trochlear grooves of the distal femurs. They were weekly respectively intra-articularly injected with: (1) 500 μL PBS; (2) 500 μL 2D-Exos suspension (1 × 10^10^ mL^−1^); (3) 500 μL 3D-Exos suspension (1 × 10^10^ mL^−1^). Four weeks later, all the animals were sacrificed with a lethal dose of anesthesia. The joints were harvested for gross appearance according to ICRS macroscopic assessment (Table 3) from three independent blinded observers. Hematoxylin and eosin (HE), Toluidine blue (TB) and safranin-O/fast green (Saf-O) were chosen as histochemistry staining. Type II collagens were performed as immunohistochemical staining. The therapeutic effects of the repaired cartilage defect were assessed by the histologic grading scale (Wakitani et al. 1994) (Table 4) from three independent blinded observers.

**Table 3:** International Cartilage Repair Society (ICRS) macroscopic assessment

Category	Points
Degree of defect repair	
In level with surrounding cartilage	4
75% repair of defect depth	3
50% repair of defect depth	2
25% repair of defect depth	1
0% repair of defect depth	0
Integration to border zone	
Complete integration with surrounding cartilage	4
Demarcating border < 1 mm	3
3/4 of graft integrated, 1/4 with a notable border > 1-mm width	2
1/2 of graft integrated with surrounding cartilage, 1/2 with a notable border > 1 mm	1
From no contact to 1/4 of graft integrated with surrounding cartilage	0
Macroscopic appearance	
Intact smooth	4
Fibrillated surface	3
Small, scattered fissures, or cracks	2
Several, small, or few but large fissure	1
Total degeneration of grafted area	0
Total maximum	12

**Table 4:** Histological grading scale for cartilage repair

Category	Points
Cell morphology	
Hyaline cartilage	0
Mostly hyaline cartilage	1
Mostly fibrocartilage	2
Mostly non-cartilage	3
Non-cartilage only	4
Matrix-staining (metachromasia)	
Normal (compared with host adjacent cartilage)	0
Slightly reduced	1
Markedly reduced	2
No metachromatic stain	3
Surface regularity	
Smooth (> 3/4)	0
Moderate (> 1/2–3/4)	1
Irregular (1/4–1/2)	2
Severely irregular (< 1/4)	3
Thickness of cartilage	
> 2/3	0
1/3–1/2	1
< 1/3	2
Integration of donor with host adjacent cartilage	
Both edges integrated	0
One edge integrated	1
Neither edge integrated	2
Total maximum	14

### Statistical analysis

The data were all showed as mean ± standard deviation (SD). Student’s *t* test or one-way ANOVA were used for comparisons among groups. *p* < 0.05 was considered as statistical significance.

## Results

### Characterization of U-MSCs and exosomes

**Fig. 1:**
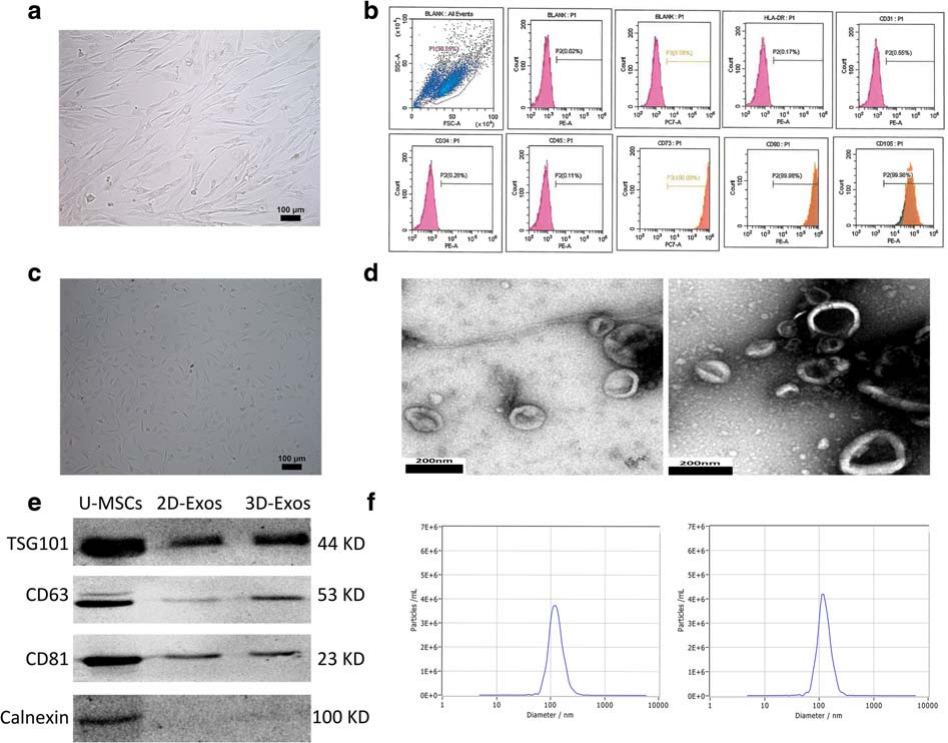
Characterization of U-MSCs and exosomes. **a** Morphological observation of U-MSCs (× 100). **b** Flow cytometric analysis of umbilical cord mesenchymal positive markers, such as CD105, CD73, and CD90, and negative markers, such as CD31, CD34, CD45, and HLA-DR. **c** Primary human chondrocyte morphology (× 100). **d** Morphology of 2D-Exos (left) and 3D-Exos (right) under transmission electron microscopy (scale bar 200 nm). **e** Western blot analysis of exosome surface markers (TSG101, CD63, CD81, and calnexin). **f** The concentration and size distribution of 2D-Exos (left) and 3D-Exos (right) by Nanosight

U-MSCs were successfully obtained from umbilical cord Wharton’s jelly. More than 95% of U-MSCs exhibited homogeneous fibroblastic morphology after three propagations (Fig. 1a). Flow cytometric analysis revealed that a majority of U-MSCs express CD105, CD73, CD90 and are negative for CD31, CD34, CD45, and HLA-DR (Fig. 1b). Primary chondrocytes were polygonal or irregular ovoid in shape, with characteristic cobblestone morphology (Fig. 1c). Transmission electron microscopy revealed a cup-shaped morphology of the 2D-Exos (Fig. 1d left) and 3D-Exos (Fig. 1d right). Western blotting revealed that the 2D-Exos and 3D-Exos express exosome-associated proteins (CD63, CD81, and TSG101) as well as negative protein (Calnexin) (Fig. 1e). Nanosight analysis demonstrated that the diameter of 2D-Exos (Fig. 1f left) and 3D-Exos (Fig. 1f right) is approximately 120 nm.

### Hollow-fiber bioreactor enables high yield production of exosomes

**Fig. 2:**
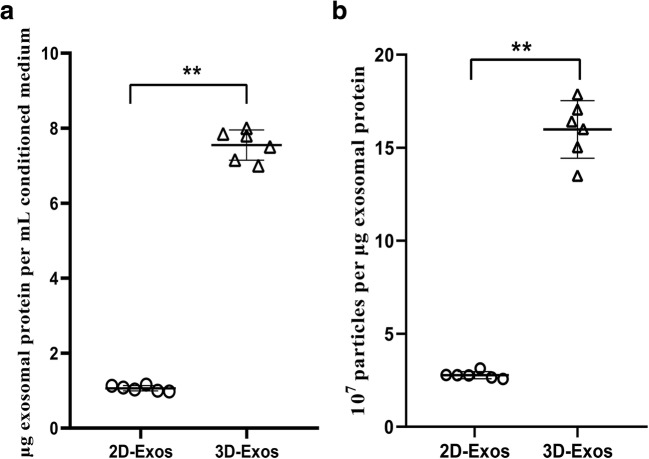
High-yield exosomes production from hollow-fiber bioreactor. **a** Yield of 3D-Exos isolated by the hollow-fiber bioreactor is ~ 7.5-fold more than conventional flask conditioned media. Protein yield = exosomal protein (μg)/original conditioned medium (mL). **b** Particle purity of 3D-Exos from the hollow-fiber bioreactor is ~ 6.7-fold higher than conventional 2D-Exos. Particle purity = the number of particles/exosomal protein (μg). Plots show yield for each method and the mean ± SD of all measurements (*p < 0.05; **p < 0.01)

The supernatants of U-MSCs cultured by the bioreactor or conventional 2D culture flask were purified for exosomes by centrifugation under identical conditions. The yield of 3D-Exos was 7.5-fold higher than that of 2D-Exos under identical conditions (Fig. 2a, Protein yield = exosomal protein (μg)/original conditioned medium (mL)). The exosome yield (μg) was determined using the Bradford assay.

The purity of exosomes was calculated from the ratio of particle to protein. The yield of 3D-Exos was approximately 1.6 × 10^8^ particles/μg of protein, which was 6.7-fold higher than that of 2D-Exos (Fig. 2b, Particle purity = number of particles/amount of exosome-associated protein (μg)). Particle purity indicates the enrichment of exosome preparations.

### Exosomes enhance proliferation and inhibit apoptosis of chondrocytes

To further validate our in vivo findings, we analyzed the underlying mechanism through the evaluation of both types of exosomes on the proliferation, anti-apoptosis, migration, and matrix synthesis of chondrocytes.

**Fig. 3:**
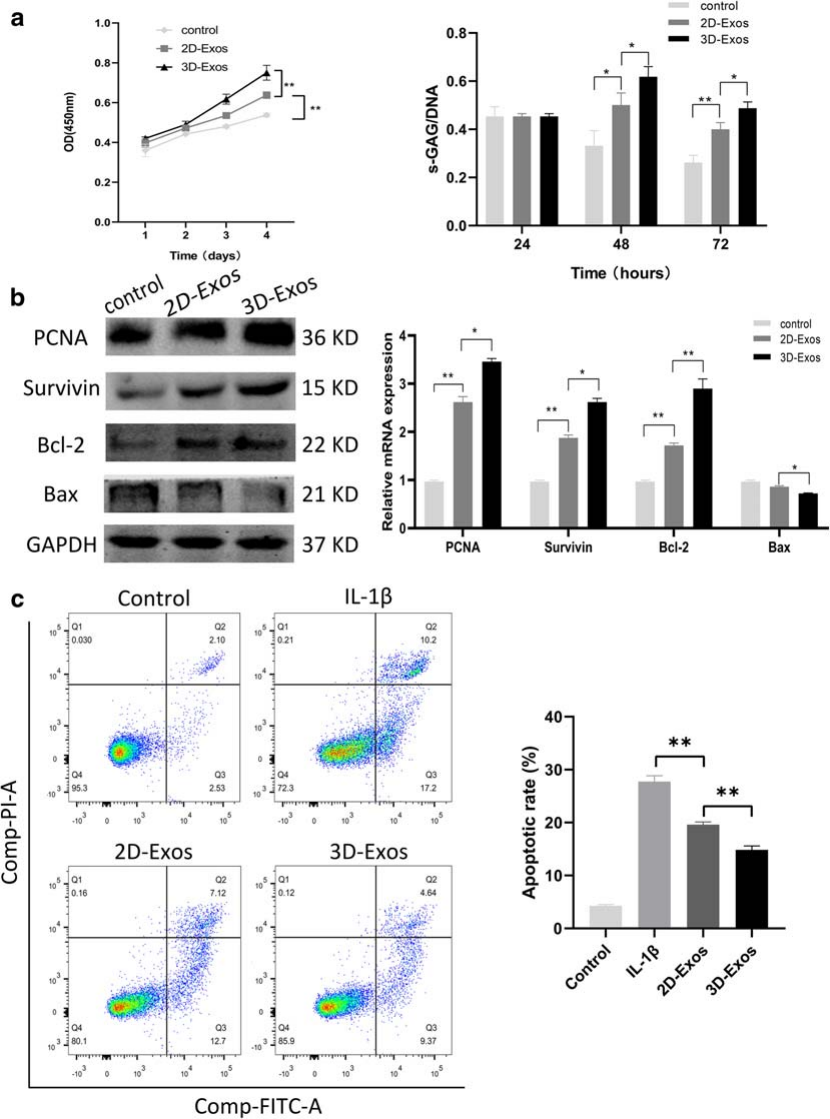
Exosomes promote proliferation and inhibit apoptosis of chondrocytes. **a** The proliferation was assessed by cck-8 assay and DNA content (right). Data represent mean ± SEM. *p < 0.05, **p < 0.01, *n* = 5. **b** The protein and mRNA level of genes associated with proliferation (PCNA) and anti-apoptosis (Survivin, Bcl-2, Bax). **c** Chondrocytes were pretreated with 2D-Exos and 3D-Exos followed by IL-1β (10 ng/mL) challenge for 24 h. Apoptosis was assessed by flow cytometry. Data represent mean ± SEM. *p < 0.05, **p < 0.01, *n* = 3

Cell proliferation was assessed using the CCK-8 assay and DNA concentration was determined. 2D-Exos and 3D-Exos were found to promote the proliferation of chondrocyte at the concentration of 10 μg/mL. Furthermore, 3D-Exos exhibited a much stronger effect on proliferation than 2D-Exos on day 4 (*p* < 0.01, Fig. 3a left). However, on day 2, there was no significant difference among the 3D-Exos, 2D-Exos, and control groups (*p* > 0.05).

The DNA concentration is proportional to the cell number. When treated with 10 μg/mL exosomes after 48 h, significant differences were observed between the exosomal and control groups (*p* < 0.05, Fig. 3a right). 3D-Exos increased the amount of DNA in cells and the results were consistent with those found in the CCK-8 assay (*p* < 0.05). The mRNA and protein expression of the genes associated with chondrocyte proliferation such as PCNA was increased within 48 h at 10 μg/mL DNA (Fig. 3b). The induction of proliferation is associated with inhibition of apoptosis. Moreover, the protein and mRNA levels of survivin, Bcl-2, and Bax were also found to be different in both groups. Next, we assessed the IL-1β-induced anti-apoptotic effect of exosomes on chondrocytes. Compared with control group (4.6 ± 0.25%), IL-1β significantly increased chondrocyte apoptosis (27.2 ± 1.1%). Importantly, pre-incubation of chondrocytes with 2D-Exos and 3D-Exos markedly reduced IL-1β-induced apoptosis to 19.6 ± 0.53% and 14.8 ± 0.76%, as shown in Fig. 3c. These results clearly demonstrate that the efficacy of 3D-Exos to inhibit chondrocyte apoptosis is significantly higher than that of 2D-Exos.

### Exosomes promote the migration of chondrocytes

**Fig. 4:**
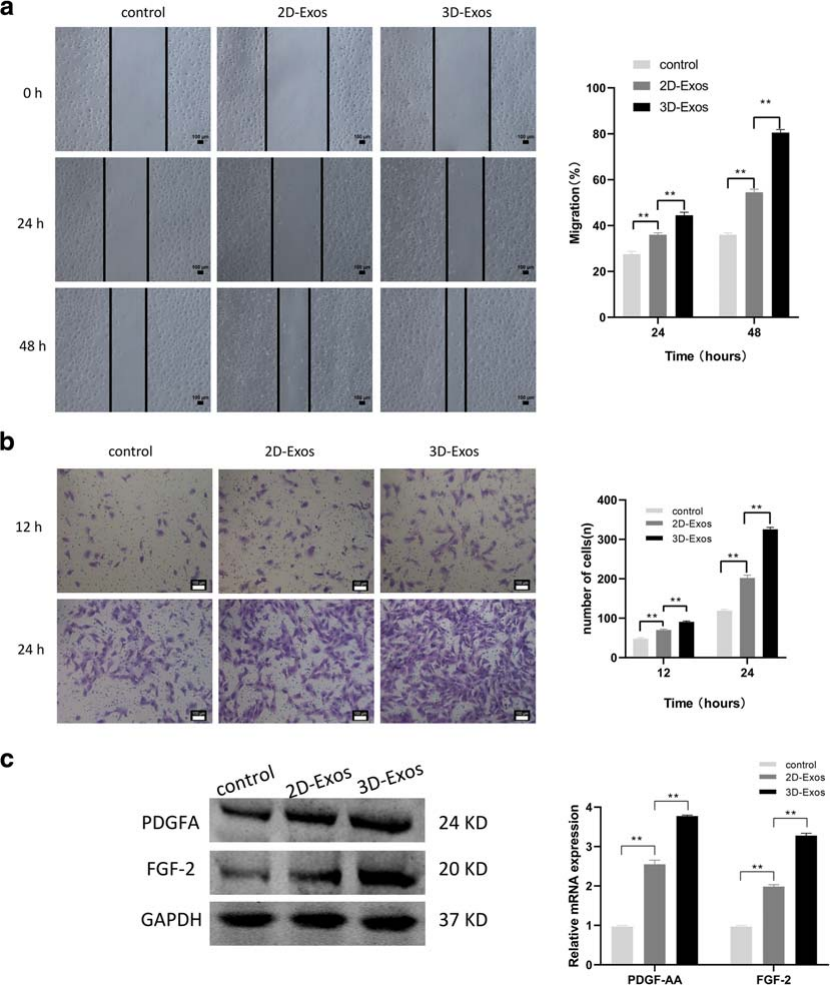
Effects of 2D-Exos and 3D-Exos on migration of chondrocytes. **a** Light microscopy images (left) and quantitative analysis (right) of scratch wound assays. **b** Light microscopy images (left) and number of transmitted cells (right) in the transwell migration assay. **c** The protein (left) and mRNA level (right) of genes associated with migration (PDGF-AA, FGF-2). Data represent mean ± SEM. *p < 0.05, **p < 0.01, *n* = 3

The wound scratch assay showed that both 2D-Exos and 3D-Exos can enhance the migration of chondrocytes at the concentration of 10 μg/mL. Moreover, 3D-Exos were more effective than 2D-Exos in promoting migration at 24 and 48 h (*p* < 0.01; Fig. 4a). The treatment of chondrocytes with 2D-Exos and 3D-Exos increased the migration rate by 2-fold to 4-fold compared to the control group. Moreover, 3D-Exos induced an approximately 2-fold higher migration rate than 2D-Exos. The transwell migration assay further proved that 3D-Exos exhibit superior exosome-stimulating effects of migration on chondrocytes than 2D-Exos (*p* < 0.01; Fig. 4b). In addition, the mRNA and protein concentrations of PDGF-AA and FGF-2—chemotactic factors of chondrocytes—were increased within 48 h at 10 μg/mL concentration (Fig. 4c).

### Exosomes promote matrix synthesis and phenotypic stability of chondrocytes

**Fig. 5:**
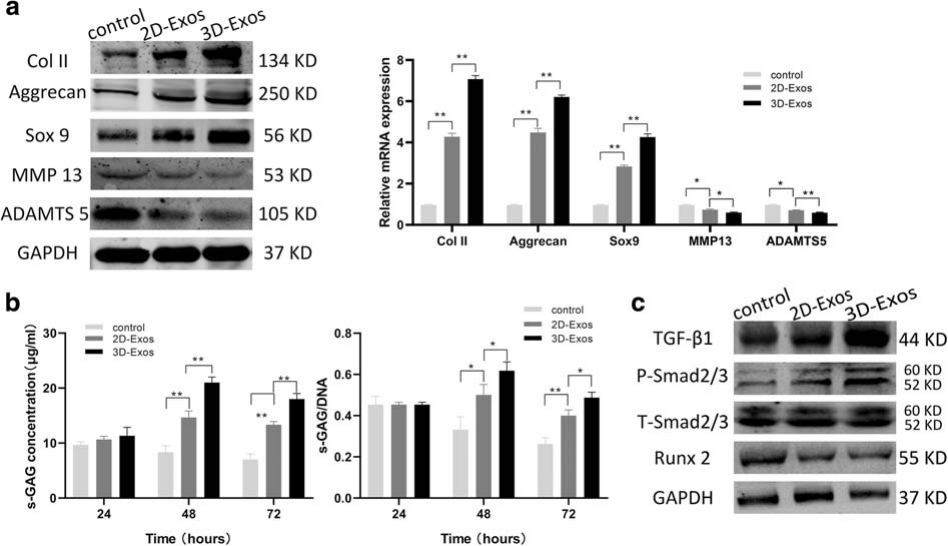
Effects of 2D-Exos and 3D-Exos on matrix synthesis and the phenotypic stability of chondrocytes. **a** The protein and mRNA level of genes associated with matrix synthesis and chondrocytic differentiation (Col II, Aggrecan, Sox 9, MMP 13, ADMST5). **b** Quantitative analysis of s-GAG (left) and s-GAG/DNA (right) when chondrocyte were stimulated by 10 μg/mL exosomes. **c** Western blot analysis of TGF-β1, P-SMAD2/3, T-SMAD2/3, and Runx 2 of chondrocytes exposed to exosomes. Data represent mean ± SEM. *p < 0.05, **p < 0.01, *n* = 3

We next explored the ability of the exosomes to maintain phenotypic stability of chondrocytes. It is noteworthy that the cartilage-specific markers—Col II, Sox9, Aggrecan—were highly upregulated by 3D-Exos; however, the hypertrophic cartilage-enriched markers ADAMTS5 and MMP 13 were downregulated by both 3D-Exos and 2D-Exos (Fig. 5a left). The mRNA levels of these proteins were consistent with the above findings (Fig. 5a right).

Along with the proliferation and passage of chondrocytes, their dedifferentiation into hypertrophic cells primarily reflects in the loss of matrix synthesis and phenotypic stability. In particular, the sulfated glycosaminoglycan (s-GAG) content was used to assess matrix synthesis of chondrocytes. At an exosomal concentration of 10 μg/mL, the contents of s-GAG and s-GAG/DNA were significantly higher at 48 h in both exosomal groups than that in the PBS group (Fig. 5b). Although the s-GAG concentration of both 2D-Exos and 3D-Exos was decreased when the incubation time was extended to 72 h, 3D-Exos showed superior ability in maintaining matrix synthesis than 2D-Exos.

These results suggest that 3D-Exos is more efficient to maintain matrix synthesis and phenotypic stability of chondrocytes than 2D-Exos.

### 3D-Exos modulate chondrocyte functions through activation of TGF-β1 and smad2/3 signaling pathways

The TGF-β1 signaling pathway is the main pathway to determine whether chondrogenesis is related to proliferation, differentiation, and matrix synthesis. Therefore, we first analyzed whether the TGF-β1-dependent Smad2/3 signaling pathway is stimulated by 3D-Exos. The protein expression of TGF-β1and phospho-Smad2/3 was increased, and that of Runx2 was decreased in vitro (Fig. 5c). Therefore, we speculated that 3D-Exos might specifically active the TGF-β1-dependent Smad2/3 signaling pathway to facilitate cartilage repair.

### Effect of exosomes on the repair of cartilage defect

**Fig. 6:**
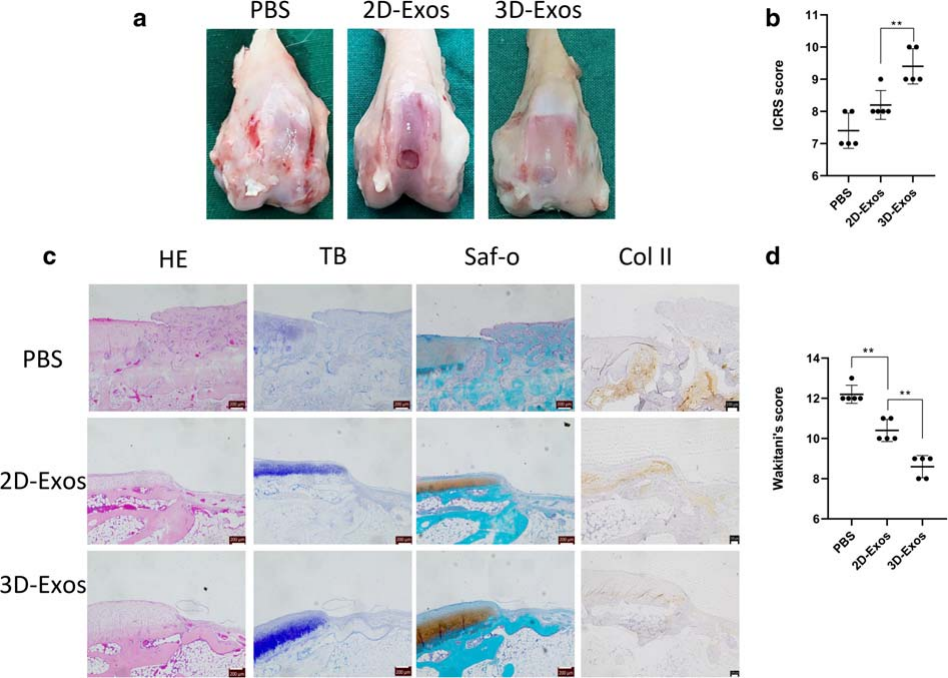
Effect of exosomes on repair of cartilage defect. **a** Representative macroscopic images of the regenerated tissues. **b** ICRS macroscopic scores. Data represent mean ± SEM. *p < 0.05, **p < 0.01, *n* = 5. **c** Staining results of HE, TB, Saf-O, and immunohistochemical staining for type II collagens. **d** Wakitani scores for the histological sections. Data represent mean ± SEM. *p < 0.05, **p < 0.01, *n* = 5

As shown in Fig. 6a, the cartilage defects treated with 3D-Exos showed more neo-tissue formation, smooth surface, and better integration with the surrounding hyaline cartilage at 4 weeks (Fig. 6a). However, the defects treated with 2D-Exos displayed poor surface regularity with structural disruptions. Moreover, the control group was the worst of all for the repair of cartilage defect as it exhibited severe tissue granulations. The ICRS macroscopic assessment scores for 3D-Exos-mediated repair and 2D-Exos-mediated repair were significantly different. However, there was no significant difference between the 2D-Exos and control groups (Fig. 6b).

Histological assessment at 4 weeks revealed that the defects treated with both types of exosomes were partly filled with hyaline cartilage characterized by chondrocytic cells (Fig. 6c). Moreover, the defects treated with 3D-Exos showed better thickness of cartilage and surface regularity than those treated with 2D-Exos. In contrast, only fibrous tissues were found in control group, and were characterized by no metachromatic stain and severe irregularity. Consistent with these observations, the 3D-Exos-treated defects exhibited the highest Wakitani score (Fig. 6d).

## Discussion

MSC-derived exosomes have been recently identified as a novel alternative to cell-based approaches in regenerative medicine. Cosenza et al. (2017) have found that the injection of exosomes derived from MSCs into the articular cavity could protect the mice from osteoarthritis. Wang et al. (2017) also demonstrated that the cartilage destruction and matrix degradation could be alleviated by exosomes derived from human embryonic MSCs in the OA mice.

Zhang et al. (2016, 2018) found that MSC-derived exosomes can effectively repair osteochondral defects by enhancing cell proliferation and infiltration, inhibiting apoptosis, and regulating immune response. Furthermore, Liu et al. (2017) used an acellular tissue patch as an exosomal scaffold to promote cartilage defect repair. These studies demonstrated that MSC-derived exosomes can be used as a promising, cell-free strategy for cartilage defect as an alternative to cell-based therapies. The study by Jarmalavičiūtė et al. (2015) demonstrated that exosomes derived from cells grown in a 3D environment show better biological functions than those derived from cells grown using the standard 2D flask culture. Thus, exosomes produced from 3D culture are not only more productive but also biologically active.

However, 3D culture has some disadvantages. Enzymatic digestion is required to retrieve exosomes produced within hydrogels; this may adversely affect exosomal integrity and bioactivity (Patel et al. 2018). Although the number of exosomes was increased, the intra-vesicular cargo content and function of exosomes were unknown. Thus, we performed this study to determine the ability of the hollow-fiber bioreactor to produce exosomes in 3D cultures and test their efficacy for cartilage repair.

In our current study, we innovatively compared the chondroprotective efficacy of exosomes derived from U-MSCs using different culture methods. The 3D culture of U-MSCs in a lab-scale hollow-fiber bioreactor enabled higher production yield (7.5-fold) and purity (6.7-fold) of exosomes, as shown in Fig. 2. In our study, we also found that exosomes derived from U-MSCs increased the number of chondrocytes and enhance their activity by stimulating cell proliferation, migration, and matrix synthesis, and inhibiting apoptosis. A series of experiments (Liu et al. 2017; Mao et al. 2018; Tao et al. 2018) were conducted to demonstrate that exosomes can significantly upregulate the mRNA and protein expression chondrogenesis-related factors, which are in agreement with our results. Furthermore, 3D-Exos exhibited a stronger effect than 2D-Exos to maintain the phenotypic stability of chondrocytes. Our preliminary data indicate that 3D-Exos might exert its effects through the TGF-β1-dependent Smad2/3 signaling pathway. Several studies have demonstrated that TGF-βs are potent inducer of chondrogenesis (Wang et al. 2016; Ying et al. 2018; Jahr et al. 2019). The protein expression of TGF-β1 and phosphorylated-Smad2/3 was increased and their downstream expression of Runx2 was decreased. Therefore, we speculated that 3D-Exos might specifically activate the TGF-β1-dependent Smad2/3 signaling pathway to facilitate cartilage repair. To our knowledge, chondrogenesis is influenced by complicated cross-talk among signaling pathways, such as Wnt, Hedgehog, BMP, and AKT/ERK (Mao et al. 2018; Ying et al. 2018; Chen et al. 2019). Further investigation is needed to picture comprehensive understanding of the pathway involved in 3D-Exos-induced chondrogenesis.

Herein, we have demonstrated that 3D-Exos exhibits more therapeutic advantages, including higher yield and enhanced function, than 2D-Exos for cartilage repair. The enhanced yield and biological function of exosomes derived from the 3D bioreactor can be clinically applied.

Although 3D-Exos showed superior effects than 2D-Exos in cartilage repair in the rabbit cartilage defect model, the therapeutic outcome was inadequate for the resumption of normal function of the cartilage. One of the reasons is that the curative time lasted only for 4 weeks; however, the actual period for cartilage repair is much longer. Therefore, we will extend the in vivo experiment to 8 or 12 weeks, or even longer to better explore the kinetics of cartilage repair. Finally, multiple injections per week were the chosen mode of administration in our study. However, this is not clinically feasible and would result in increased pain to the patients. Moreover, local injection of exosomes into the joint space cannot guarantee their retention in the defect site. This would eventually lead to uncertain bio-distribution of the exosomes. Therefore, exosomes embedded in scaffolds such as hydrogel tissue patch could be explored to effectively cover the cartilage defect.

Our findings indicate that culture conditions not only affect the functional status of cells but also affect exosomal activity and property. The difference in the activities and properties of 2D-Exos and 3D-Exos is related to the differences in their cargo content. We speculate that a myriad of components such as microRNAs or proteins are present in 3D-Exos, and play a crucial role in orchestrating cartilage regeneration, including promoting cell proliferation, migration, matrix synthesis, inhibiting apoptosis, and maintaining phenotypic stability of chondrocytes. However, the mechanism by which 3D-Exos exhibit superior curative effect than 2D-Exos is still unclear. Subsequent studies will be focused on comparative proteomic and RNA-seq analyses to dissect the proteins and microRNAs that are responsible for the beneficial effects. These studies will provide insights into the mechanisms underlying osteochondral regeneration of exosomes derived from 3D culture.

## Conclusion

In summary, our study provides novel insights into the chondroprotective properties of exosomes derived from the 3D culture of U-MSCs in a hollow-fiber bioreactor. More specifically, 3D-Exos increased the number and enhance the function of chondrocytes by stimulating cell proliferation, migration, and matrix synthesis, and inhibiting apoptosis. Because of its enhanced biological function and yield, 3D-Exos may represent a promising therapeutic method for the treatment of cartilage defects.
